# 3D Printer Generated Tissue iMolds for Cleared Tissue Using Single- and Multi-Photon Microscopy for Deep Tissue Evaluation

**DOI:** 10.1186/s12575-017-0057-2

**Published:** 2017-07-05

**Authors:** Sean J. Miller, Jeffrey D. Rothstein

**Affiliations:** 1 Department of Neurology, Johns Hopkins University School of Medicine, Johns Hopkins Univ., 855 N Wolfe Street, Room 250.18, Baltimore, MD 21205 USA; 2 The Brain Science Institute at Johns Hopkins, Johns Hopkins Univ. School of Medicine, Johns Hopkins Univ., 855 N Wolfe Street, Baltimore, MD 21205 USA; 3 Cellular and Molecular Medicine Johns Hopkins School of Medicine, Johns Hopkins Univ., 855 N Wolfe Street, Room 250.18, Baltimore, MD 21205 USA

**Keywords:** CLARITY, Tissue molds, Microscopy, Cleared tissue, 3D printer, Medicine

## Abstract

**Background:**

Pathological analyses and methodology has recently undergone a dramatic revolution. With the creation of tissue clearing methods such as CLARITY and CUBIC, groups can now achieve complete transparency in tissue samples in nano-porous hydrogels. Cleared tissue is then imagined in a semi-aqueous medium that matches the refractive index of the objective being used. However, one major challenge is the ability to control tissue movement during imaging and to relocate precise locations post sequential clearing and re-staining.

**Methods:**

Using 3D printers, we designed tissue molds that fit precisely around the specimen being imaged. First, images are taken of the specimen, followed by importing and design of a structural mold, then printed with affordable plastics by a 3D printer.

**Results:**

With our novel design, we have innovated tissue molds called innovative molds (iMolds) that can be generated in any laboratory and are customized for any organ, tissue, or bone matter being imaged. Furthermore, the inexpensive and reusable tissue molds are made compatible for any microscope such as single and multi-photon confocal with varying stage dimensions. Excitingly, iMolds can also be generated to hold multiple organs in one mold, making reconstruction and imaging much easier.

**Conclusions:**

Taken together, with iMolds it is now possible to image cleared tissue in clearing medium while limiting movement and being able to relocate precise anatomical and cellular locations on sequential imaging events in any basic laboratory. This system provides great potential for screening widespread effects of therapeutics and disease across entire organ systems.

**Electronic supplementary material:**

The online version of this article (doi:10.1186/s12575-017-0057-2) contains supplementary material, which is available to authorized users.

## Background

Histology is a technique focused on evaluating tissue sections in many different elements. Historically, in basic science laboratories groups would undergo tremendous efforts with high room for error to obtain histological samples ready for imaging. To resolve this issue, groups have recently developed tissue clearing techniques that render samples transparent and intact [1–3]. This advancement in histology has allowed groups to visualize whole anatomical specimens with 3D reconstructions and analyses. Additionally, cleared tissue in hydrogel complexes are capable of antibody removal and re-staining; something infeasible in prior methods [1]. However, one major limitation is the ability to limit tissue movement and to relocate exact anatomical locations post re-staining and mounting in tissue clearing medium. This is especially important for the sequential evaluation of multiple biological markers.

With current imaging setups for cleared tissue, the tissue is held by a semi-hardened material such as putty. The tissue is placed within the putty prior to and during imaging. Post imaging, the putty is removed as well as the tissue. For the sequential imaging, new putty has to be used to hold the tissue. However, this leaves the tissue in unprecise locations in each imaging session making it challenging for users to relocate exact biological targets of interest. Furthermore, if the user is attempting to re-image multiple organs at once on sequential stainings, then it is near impossible to place the tissue in the exact locations from the original imaging.

The need for a device that can securely hold any tissue sample in a refined position with the tissue sample capable of being removed and replaced in the same position is of high importance. Not only would this allow groups to relocate areas of interest with sequential stains, it would also allow users to easily set imaging parameters that don’t require the user to be present during the imaging session (by pre-setting the microscope coordinates). Thus, reducing movement, improving re-evaluations of biological targets after re-staining, and saving time compared to the other methodologies.

To develop a device that could provide these improvements we chose to generate tissue specific molds, called innovative molds (iMolds). iMolds are printed by inexpensive 3D printers in several different plastics that can be sterilized and are generated specific to the tissue specimen it is supporting. Overall the plastic materials and 3D printers are dramatically cheaper than using excess clearing solution, iSpacers, and other materials on the market. Lastly, iMolds allow users to evaluate and re-evaluate multiple organs in one imaging session.

## Methods

### Materials and Reagents

Polylactic acid (PLA) plastic was purchased from micro3D (USA); low magnification tissue images were obtained with an iPhone 6S from Apple Inc. (USA); 32% Paraformaldehyde (PFA) purchased from Electron Microscopy Sciences (USA); 10X PBS from Quality Biological Inc. (USA); Tissue clearing solution from Logos Biosystems (USA); Photoinitiator VA-044 from Wako chemicals (Germany); Histodenz from Sigma (USA).

### iMold Engineering Software

.STL files were generated in Google Sketchup Software; Prototypes were printed using the Micro 3D printing software.

### 3D Printer

M3D printer was purchased from micro3D (USA); all printing parameters were not adjusted from the manufacturer’s settings.

### Tissue Clearing

To achieve transparent tissue in organs and bones we followed the protocol previously established [4].

### Tissue Staining

For deep tissue staining we followed the protocol previously established for 5 days with primary antibody and three days for secondary antibody [4].

### Sterilization

Tissue iMolds can be placed under UV light to sterilize, we recommend a duration of at least one hour [5].

## Results

### Generation of the Tissue iMold

**Fig. 1:**
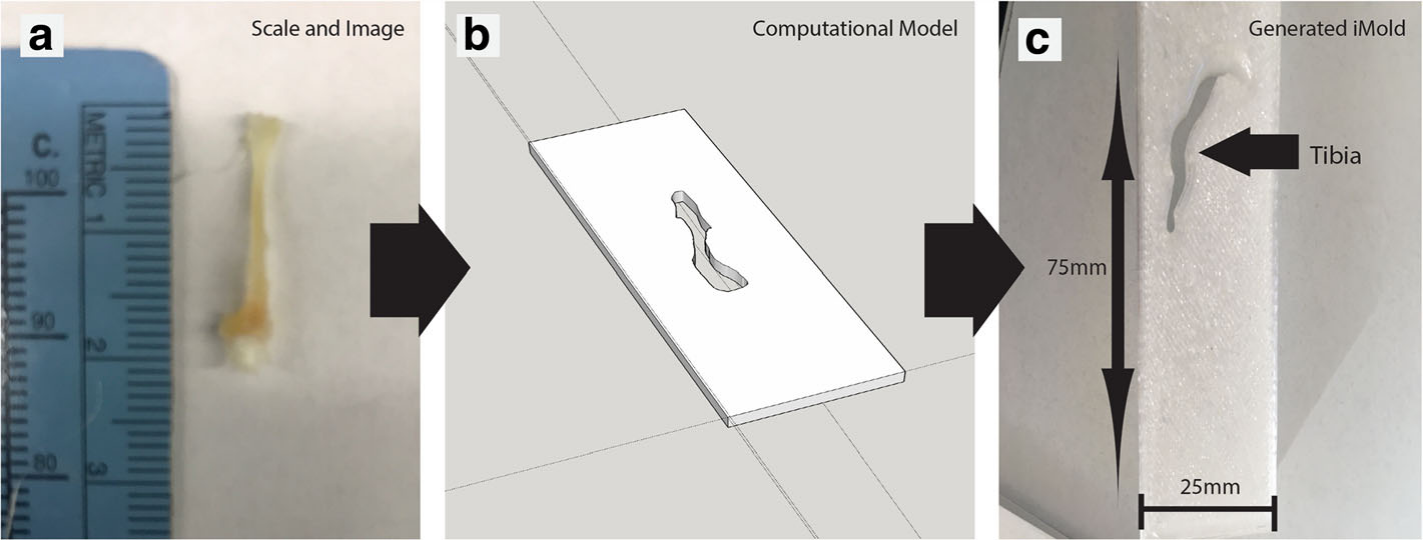
Generation and imaging with tissue iMolds. **a** a photo of the tissue (tibia) to be cleared next to a ruler, **b** generation of a slide base with the tissue (tibia) inserted inside, **c** 3D printed tissue iMold for tibia

To begin the process of generating a tissue mold that is specific to the tissue specimen being analyzed, the user must take a photo of the tissue being prepared for clearing and iMold generation, and place the tissue next to a ruler or scale bar (Fig. 1a). Taking a photo prior to tissue clearing is preferred because once the tissue has cleared it becomes very challenging to generate iMolds due to the tissue transparency. Next, the image is imported into Google Sketchup or another 3D printer software to generate an.STL file. The Google Sketchup scale is then set to the appropriate scale bar as the photo taken with the tissue (i.e. millimeters, inches) (Fig. 1a). Next the user will precisely trace the outside shape of the tissue specimen (this can be performed in other software packages like Adobe Photoshop). After successfully tracing the tissue specimen, delete the original image, keeping only the new traced image.

Next generate the slide base that will be used on the microscope, this is generally the same size as a slide or a cell culture plate. Thus, this slide base can range from the size of a whole plate or to a typically slide size (i.e. 25 mm × 75 mm); this is determined by your imaging platform. Once the slide base is generated place the traced tissue specimen shape into the desired location in the slide base on the same Z-plane in the software.

Now, once again remove the inside of the traced tissue that has been just placed within the slide base. This will now appear as a slide base with a missing tissue specimen shape (Fig. 1b). Next raise (i.e. pull) the slide specimen to the appropriate height measured during the initial step (i.e. the height of the tissue). Now you have successfully generated an iMold design. Now, export the file as a.STL for use in the 3D printer. Finally, transfer and print the 3D mold.STL file with the 3D printer (Fig. 1c). Parameters used with the micro3D software included no raft or support material, as this would have to be removed post printing, and a print quality of 150 μm with fill density selected as high. Once you have imported the.STL file and set the parameters correctly, then it is now ready to be printed. Prior to using the finished iMold, super glue a piece of cover glass to one side of the iMold. If you plan to image from both sides, then coverglass needs to be placed on both sides. After imaging the coverglass can be removed (if double sided) with the use of forceps. However, this can result in the glass breaking, in which the user would have to reprint the saved.STL file. Due to this, we highly recommend only imaging from one side therefore the coverglass will not have to be removed when using an inverted microscope.

### Preparing for Imaging on a Single or Multi-Photon Confocal System

Next, it is time for imaging on single or multi-photon confocal systems. Place the cleared organ into the iMold (it should fit appropriately to inhibit movement) and with a pipet fill the iMold with clearing solution for imaging. If the size is too small or too large, you may go back to the original Google Sketchup file and expand or contract the iMold. Now find the starting point of the tissue being imaged and z-stack tiled. Set the coordinates for tiling the entire tissue or the area of interest and begin. Once you retrieve the image, there should be no movement from the tissue.

**Fig. 2:**
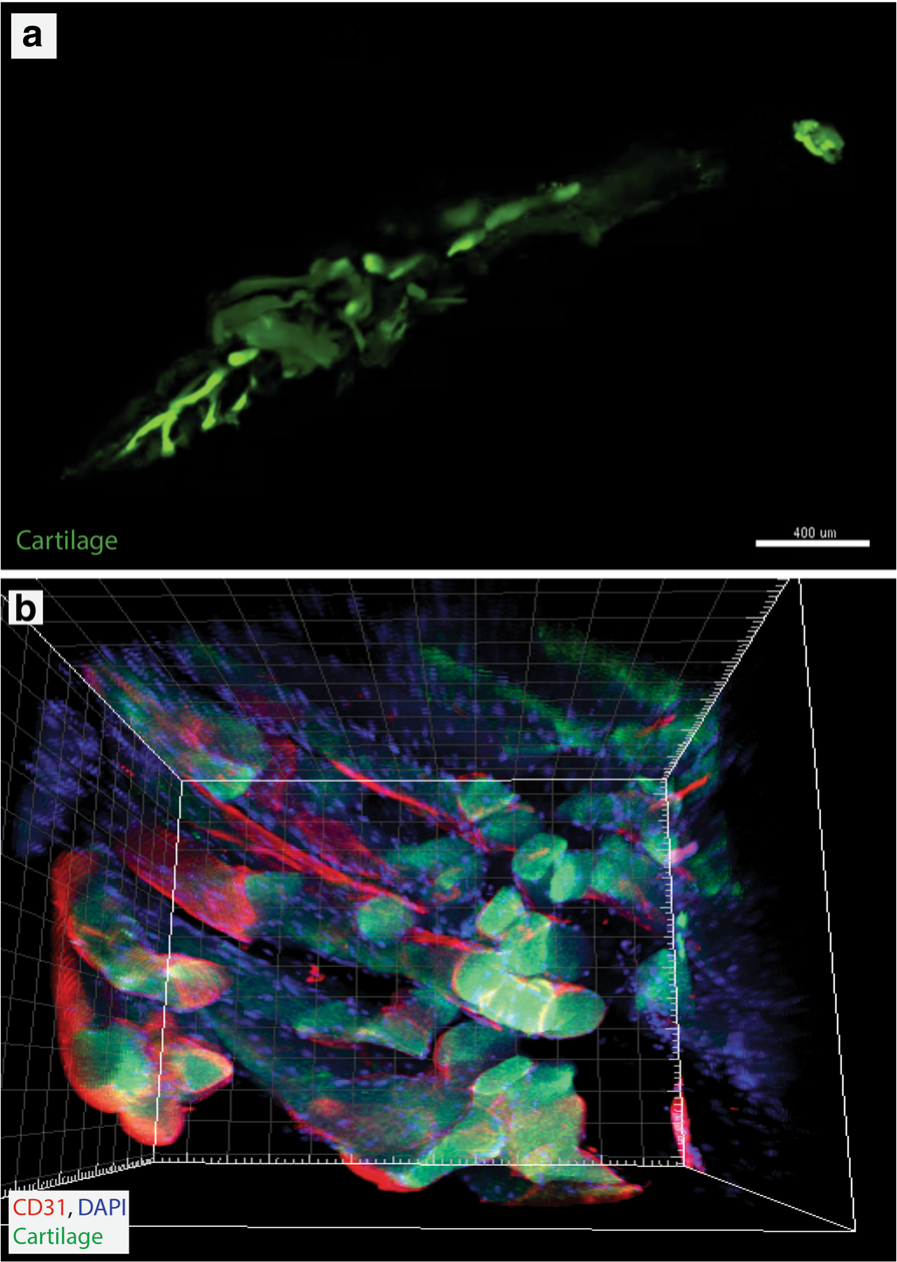
Imaging with iMolds. **a** imaging of the tissue (tibia) in the iMold, **b** Imaging of the metaphysis vasculature and bone

We chose to use the tibia for our demonstration. Bone is notoriously challenging to clear due to low lipid concentrations, undergoes immense amounts of movement while imaging, and therefore we wanted to show both clearing of whole bone and the end results of the tibia in the iMold (Fig. 2) [6]. After z-stack tiling the entire tibia in the iMold we were capable of reconstructing the tissue with internal bone and cartilage structures on the 488 nM wavelength, using a Zeiss LSM 800 confocal, and Bitplane Imaris software suite (Fig. 2a, Additional file 1: Video 1) [7]. Simply import the image file (e.g. czi) into Imaris and use the automatic 3d rendering for visualization. To relocate exact areas after sequential imaging sessions we recommend starting to image from a set coordinate (e.g. corner of the iMold) or by placing a dot with a marker onto the glass and use the same Z-stack tiling parameters on the microscope. Next, we stained the tibia with marker CD31 to visualize areas of blood vessels, using the previously published protocol [4]. We found, in support of prior literature, that the cartilage of the metaphysis and the vasculature closely interact, within a few microns of each other (Fig. 2b) [8].

Lastly, we generated an iMold that can fit multiple organs into the mold, such as brain and bone to emphasize the user flexibility and ability to customize the iMold for imaging (Additional file 2: Figure S1). This multiple organ iMold allows groups to evaluate multiple organs and/or bones without the need for individual molds.

## Discussion

The revolution of imaging cleared tissue is allowing groups to finally image whole organs at super resolution with multiple targets of interest stained [7]. For therapeutic studies that have an unknown effect on other organs, this system is an ideal way to rapidly scan through multiple organs. In neuroscience, it has been used to identify and follow neuronal projections throughout the central nervous system furthering our understanding of neuronal communications. However, there are no current devices that allow you to place organs of interest into pre-casted molds for repeated imaging. Another caveat to imaging cleared tissue is the usage of semi-aqueous solutions during imaging sessions which allows the organ to move throughout scans and challenging to relocate areas of interest during sequential imaging events.

To address the issues with the current imaging modality of cleared tissue and to provide additional benefits we have successfully generated tissue molds, iMolds. iMolds for cleared tissue limits the overall movement, can be reused and allow for exact areas to be re-analyzed post sequential staining, and are very inexpensive for any basic science lab to produce. These provide essential improvements to prior methods [9]. iMolds are user customized allowing for multiple organs in one mold or abnormal tissue architecture such as tumors to be imaged, in addition to usage on any stage for single and multi-photon confocals. Future studies will focus on advancing iMolds for use in light sheet microscopy where samples are typically submerged in an aqueous solution during imaging [10]. In closing, we strongly believe that the open access to generating iMolds will allow groups to significantly improve their imaging quality and exploration of whole organs.

## Conclusion

Generating a device to repeatedly scan biological specimens in the exact locations is essential for repeated imaging modalities. With the advantage of iMolds over current methodologies, we believe that this novel design will speed up imaging and processing of cleared tissue making CLARITY and other clearing techniques more feasible for basic science labs and drug screening.

## Additional files

Additional file 1: Video representation of a 3d rendering of the cleared tibia in the tissue iMold. (MOV 6251 kb)

Additional file 2: Figue S1. iMolds can hold multiple organs per mold. A) iMold for brain and tibia in one slide base. (TIFF 7721 kb)

## Abbreviations

iMold: innovative mold

## References

[1] Chung K (2013). Structural and molecular interrogation of intact biological systems. Nature.

[2] Susaki EA (2014). Whole-brain imaging with single-cell resolution using chemical cocktails and computational analysi*s*. Cell.

[3] Renier N (2014). iDISCO: a simple, rapid method to immunolabel large tissue samples for volume imaging. Cell.

[4] Yang B (2014). Single-cell phenotyping within transparent intact tissue through whole-body clearing. Cell.

[5] Valente TAM (2016). Effect of sterilization methods on electrospun poly(lactic acid) (PLA) fiber alignment for biomedical applications. ACS Applied Materials Interfaces.

[6] Acar M (2015). Deep imaging of bone marrow shows non-dividing stem cells are mainly perisinusoidal. Nature.

[7] Miller SJ, Rothstein JD (2016). Astroglia in thick tissue with super resolution and cellular reconstruction. PLoS One.

[8] Lo Celso C (2009). Live-animal tracking of individual haematopoietic stem/progenitor cells in their niche. Nature.

[9] Rapid, simple and inexpensive production of custom 3D printer equipment for large-volume fluorescence microscopy*.* Int J Pharm. 2015;494(2): p. 651–656. DOI: 10.1016/j.ijpharm.2015.03.042. 10.1016/j.ijpharm.2015.03.042 PMC4626572 25797056

[10] Schmied C, Tomancak P (2016). Sample preparation and mounting of drosophila embryos for Multiview light sheet microscopy. Methods Mol Biol.

