# Supplementary figures and images for: 3D Printer Generated Tissue iMolds for Cleared Tissue Using Single- and Multi-Photon Microscopy for Deep Tissue Evaluation
Source: Biol Proced Online. 2017 Jul 5;19:7. doi: 10.1186/s12575-017-0057-2 (PMC5497344; doi:10.1186/s12575-017-0057-2)

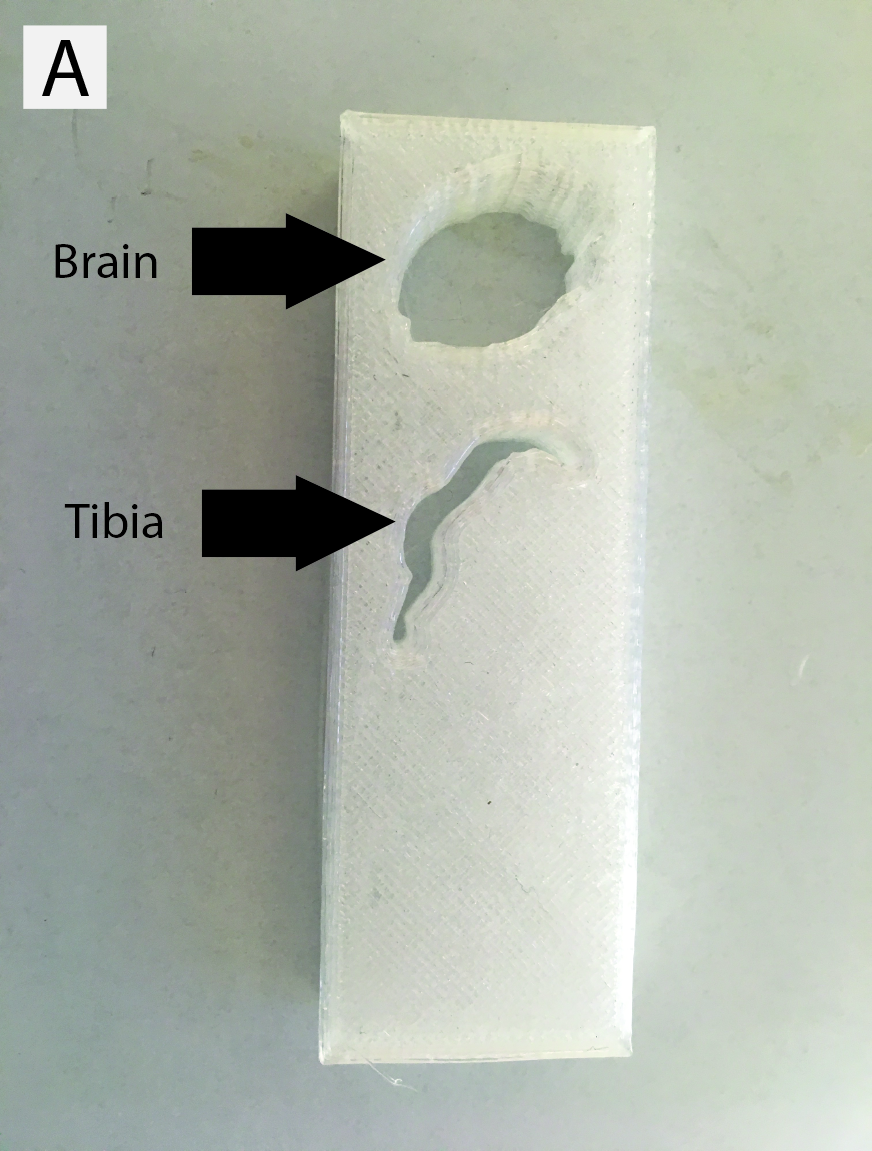

Supplement: Supplementary file 2 — iMolds can hold multiple organs per mold. A) iMold for brain and tibia in one slide base. (TIFF 7721 kb) [file 12575_2017_57_MOESM2_ESM.tif]
